# Tulp3 deficiency results in ciliopathy phenotypes during zebrafish embryogenesis

**DOI:** 10.1038/s41598-025-16584-3

**Published:** 2025-09-12

**Authors:** Daniel Epting, John Devane, Ralf Mertes, Séverine Kayser, Martin Helmstädter, Patrick Metzger, Melanie Boerries, Carsten Bergmann, Elisabeth Ott

**Affiliations:** 1 Department of Medicine IV, Faculty of Medicine, Medical Center-University of Freiburg, University of Freiburg, Freiburg, Germany; 2 Institute of Medical Bioinformatics and Systems Medicine, Faculty of Medicine, Medical Center-University of Freiburg, University of Freiburg, Freiburg, Germany; 3 Faculty of Biology, University of Freiburg, Freiburg, Germany; 4 Faculty of Medicine, University of Freiburg, Freiburg, Germany; 5 German Cancer Consortium (DKTK), Partner site Freiburg, DKFZ and Medical Center-University of Freiburg, Freiburg, Germany; 6 Medizinische Genetik Mainz, Limbach Genetics, Mainz, Germany

**Keywords:** Cell biology, Computational biology and bioinformatics, Developmental biology, Genetics, Molecular biology, Nephrology

## Abstract

Ciliopathies, caused by defective cilia biogenesis or function, comprise a genetically and clinically diverse group of diseases. Primary cilia play pivotal roles in the regulation of a multitude of signalling pathways during development and tissue homeostasis. Cilia assembly, maintenance and signalling depend on intraflagellar transport (IFT). Tubby-like protein 3 (TULP3) functions as an adapter protein for the ciliary trafficking of diverse membrane cargos via an interaction with the IFT-A complex. Recently, we and others have shown that individuals carrying pathogenic TULP3 variants suffer from progressive liver, kidney and heart disease. In line with these findings, adult Tulp3 knockout zebrafish displayed liver fibrosis and kidney cyst phenotypes. In the present study, we analysed the functional consequences of Tulp3 deficiency during zebrafish embryogenesis. Tulp3 deficiency resulted in well-known ciliopathy-associated phenotypes including pronephric cysts, body curvature and altered left-right asymmetry. Our analysis of urotensin 2-related peptide (Urp) signalling, which is required for proper spine morphogenesis, revealed reduced expression of *urp1* in Tulp3 knockout embryos. We also observed scoliosis in a significant number of adult Tulp3 knockout zebrafish. Analysis of ciliogenesis revealed a reduced cilia number and ciliary length in Tulp3 deficient embryos. In addition, Tulp3 deficiency resulted in upregulation of cilia-dependent profibrotic Wnt and Jak/Stat signalling components. Furthermore, we demonstrate that loss of Tulp3 causes upregulation of genes related to liver fibrosis. In conclusion, our data highlights a role of Tulp3 in proper cilia formation and function to maintain healthy tissue architecture during zebrafish embryogenesis, and provides further insight into the spectrum of cilia-related phenotypes in adult zebrafish depleted for Tulp3 functions.

**Supplementary Information:**

The online version contains supplementary material available at 10.1038/s41598-025-16584-3.

## Introduction

Primary cilia, microtubule-based sensory organelles protruding from the apical surface of almost all vertebrate cells, play key roles in cell signal transduction and thereby in development and tissue homeostasis^1–4^. Defects in the biogenesis, maintenance or function of cilia lead to a large group of inherited diseases, collectively termed ciliopathies^5–7^. Ciliopathies are characterized by the presence of renal cysts, but can also manifest in other organs including the brain, liver, pancreas, heart and eye. Frequently, respective clinical management is challenged by diagnostic ambiguity, variable disease expression and incomplete penetrance^8–10^. Therefore, disease classification highly depends on a profound understanding of the molecular mechanisms that are affected. Furthermore, animal models provide us with the opportunity to follow disease progression from embryogenesis to adulthood, and thereby offer essential information in disease development^11^. Over the last two decades the ciliary transition zone (TZ) has gained specific interest because many disease-causing genes have been localized to this evolutionary conserved subdomain at the proximal end of the ciliary axoneme^6,12^. The ciliary TZ regulates the membrane specific distribution of certain phospholipids, and is thereby attributed a diffusion barrier function^13,14^. In vitro and in vivo models showed that Tubby-like protein 3 (TULP3) acts as a critical adapter protein for the ciliary trafficking of transmembrane proteins (e.g., polycystins, fibrocystin and a subset of G protein-coupled receptors (GPCRs)), and membrane-associated proteins (e.g., ADP-ribosylation factor-like protein 13B (ARL13B) and inositol polyphosphate-5-phosphatase E (INPP5E))^15–20^. TULP3-dependent transport of proteins to the ciliary membrane requires its binding to the intraflagellar transport protein complex A (IFT-A) and phosphoinositide 4,5-bisphosphate (PI(4,5)P_2_)^16,18,21^. While PI(4,5)P_2_ is restricted to the ciliary base, PI(4)P localizes to the ciliary axoneme^22,23^. Loss of the Joubert syndrome-associated INPP5E leads to mislocalization of PI(4,5)P_2_ along the cilium. Subsequently, the disruption of the ciliary barrier function results in accumulation of TULP3 and its binding partner GPR161 to the ciliary membrane^24,25^. Homozygous loss of TULP3 in mice causes defective neural tube closure, polydactyly and eventually results in embryonic lethality^26^. Furthermore, *Tulp3* knockout mice display an expansion in Hedgehog (HH) signalling related gene expression revealing that TULP3 functions as a negative regulator of the HH signalling pathway^27–29^. Mice carrying a hypomorph missense mutation in *Tulp3* develop late embryonic kidney cysts, skeletal deformities and neural patterning defects^17^. While cilia formation and morphology in these mice are not affected, the ciliary axoneme transport of Polycystin-2 and ARL13B is reduced. In individuals affected by progressive degenerative liver fibrosis, fibrocystic kidney disease and hypertrophic cardiomyopathy, we and others have previously identified bi-allelic deleterious variants in *TULP3*^30,31^. Ciliary trafficking of TULP3 cargo proteins was significantly reduced in TULP3 patient derived cells. While previous reports demonstrated a negative regulatory function of TULP3 on HH signalling, in a patient carrying a hypomorph mutation in TULP3 we found increased wingless/integrated (WNT) and Janus kinase/signal transduction and transcription activation (JAK/STAT) signalling^27,28,30^. Notably, recent research indicates that dysfunctional cilia act as important mediators of fibrosis in various tissues and organs via upregulation of distinct signalling pathways (HH, WNT, TGFβ, NOTCH and JAK/STAT signalling)^32–39^.

To study TULP3 in vivo functions in greater detail, we here used the zebrafish as a vertebrate model organism. We show that the knockdown of Tulp3 resulted in well-known ciliopathy-related phenotypes as well as defective ciliogenesis during zebrafish embryogenesis^40^. We could further validate these embryonic phenotypes in a previously generated CRISPR/Cas9-induced maternal-zygotic (MZ) *tulp3* knockout zebrafish line^30^. We have recently shown that homozygous *tulp3* knockout zebrafish developed into adulthood, but in this phase developed liver fibrosis and kidney cysts, a phenomenon similar to that observed in human patient phenotypes^30^. We now additionally documented spinal deformity in adult *tulp3* mutant zebrafish. Our analysis in MZ*tulp3* embryos revealed no obvious defects in the formation of the cilia-dependent Reissner fiber (RF), a structure that is implicated in proper body axis morphogenesis in zebrafish^41,42^. However, we identified dysregulation of RF-induced expression of *urotensin 2-related peptide 1* (*urp1*) in ventral cerebrospinal fluid contacting neurons (CSF-cNs) in MZ*tulp3* embryos, encoding for a neuropeptide that is involved in zebrafish axis straightness^43–49^. Our expression analyses of various cilia-related signalling pathway components revealed upregulation of profibrotic Wnt and Jak/Stat signalling, while Hh signalling was unaffected. Furthermore, in Tulp3 depleted embryos we observed upregulation of genes related to liver fibrosis^50^. Altogether, we here show that the loss of Tulp3 causes defects in ciliary function during early zebrafish development that eventually lead to liver fibrosis, cystic kidney disease and scoliosis in adult animals.

## Results

### Knockdown of Tulp3 results in ciliopathy-associated phenotypes during zebrafish embryogenesis

**Fig. 1:**
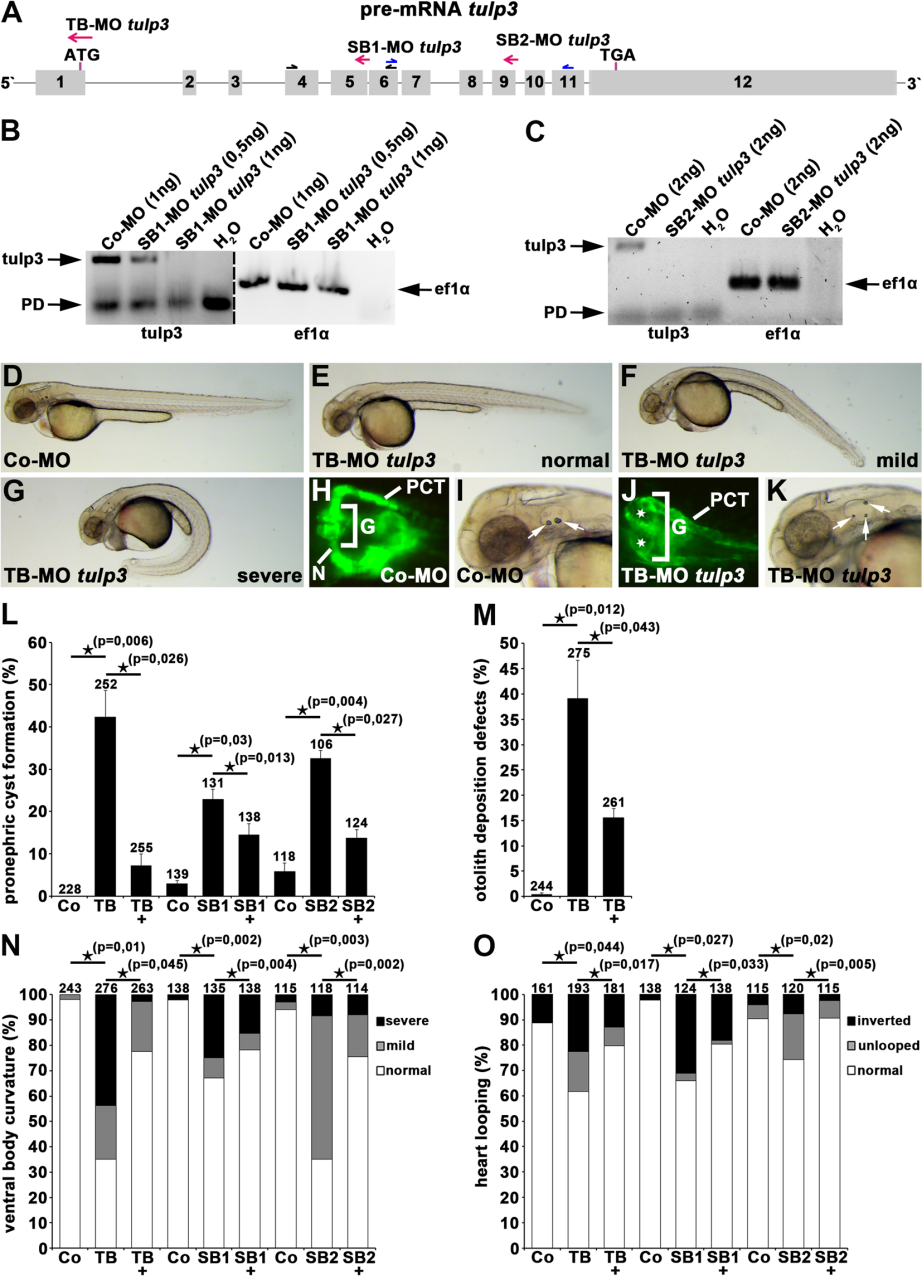
Tulp3 knockdown analyses of cilia-related phenotypes during zebrafish embryogenesis. **(A)** Exon-intron structure (drawn to scale) of *tulp3* in zebrafish (Ensembl Transcript ID: ENSDART00000093236.6). Translation start codon (ATG), termination codon (TGA), TB-MO *tulp3*, SB1-MO *tulp3* and SB2-MO *tulp3* are indicated. Black and blue half arrows indicate primer pairs used for analysis of SB1-MO *tulp3* and SB2-MO *tulp3* efficiency, respectively. **(B**,** C)** Expression analysis of *tulp3* using semi-quantitative RT-PCR on cDNA of Co-MO (1ng) or SB1-MO *tulp3* (0,5 or 1ng) injected embryos **(B)** and Co-MO (2ng) or SB2-MO *tulp3* (2ng) injected embryos, respectively **(C)**; while an exon exclusion is not detectable upon injection of SB1-MO *tulp3* or SB2-MO *tulp3*, respectively, the highly reduced wildtype PCR products compared to the control indicate an (partial) intron insertion through cryptic splice site activation that might not be detectable by utilized PCR conditions. Black arrows point to tulp3 and ef1α PCR products, respectively; Primer dimer (PD). H_2_O served as negative control and ef1α as loading control; dividing lines in **(B)** indicate different contrast from different parts of the same gel image. **(D-G)** Bright-field images of zebrafish embryos at 2dpf injected with Co-MO (2ng) **(D)** or TB-MO *tulp3* (2ng) **(E-G)**. In comparison to Co-MO injected embryos, injection of TB-MO *tulp3* leads to different degrees of ventral body curvature. **(H–K)** Knockdown of Tulp3 leads to pronephric cyst formation (white stars in **(J)**) and otolith deposition defects (white arrows in **(K)**) at 2dpf as shown in a dorsal view with anterior to the left of a TB-MO *tulp3* (2ng) injected *li1Tg* embryo **(J)**, and an embryo shown in a bright-field image **(K)**, respectively, in comparison to Co-MO (2ng) injected embryos **(H**,** I)**; expression of EGFP fluorescence labels glomerulus (G), neck (N) and proximal convoluted tubule (PCT). **(L)** Quantification of pronephric cyst formation in 2dpf zebrafish embryos injected with Co-MO (2ng) (4 independent experiments; *n* = 31, *n* = 63, *n* = 52 and *n* = 82 analysed embryos, respectively), TB-MO *tulp3* (2ng) (*n* = 35, *n* = 79, *n* = 57 and *n* = 81), TB-MO *tulp3* (2ng) + *HTULP3* mRNA (5pg) (*n* = 54, *n* = 71, *n* = 57 and *n* = 73) or Co-MO (0,5 ng) (3 independent experiments; *n* = 46, *n* = 46 and *n* = 47), SB1-MO *tulp3* (0,5 ng) (*n* = 41, *n* = 44 and *n* = 46), SB1-MO *tulp3* (0,5 ng) + *HTULP3* mRNA (5pg) (*n* = 46, *n* = 46 and *n* = 46) or Co-MO (2ng) (3 independent experiments; *n* = 42, *n* = 39 and *n* = 37), SB2-MO *tulp3* (2ng) (*n* = 31, *n* = 37 and *n* = 38), SB2-MO *tulp3* (2ng) + *HTULP3* mRNA (5pg) (*n* = 41, *n* = 41 and *n* = 42). **(M)** Quantification of otolith deposition defects in 2dpf zebrafish embryos injected with Co-MO (2ng) (4 independent experiments; *n* = 42, *n* = 70, *n* = 50 and *n* = 82 analysed embryos, respectively), TB-MO *tulp3* (2ng) (*n* = 44, *n* = 79, *n* = 71 and *n* = 81), TB-MO *tulp3* (2ng) + *HTULP3* mRNA (5pg) (*n* = 56, *n* = 71, *n* = 56 and *n* = 78). **(N)** Quantification of ventral body curvature in 2dpf zebrafish embryos injected with Co-MO (2ng) (4 independent experiments; *n* = 41, *n* = 70, *n* = 50 and *n* = 82 analysed embryos, respectively), TB-MO *tulp3* (2ng) (*n* = 40, *n* = 80, *n* = 72 and *n* = 84), TB-MO *tulp3* (2ng) + *HTULP3* mRNA (5pg) (*n* = 57, *n* = 71, *n* = 56 and *n* = 79) or Co-MO (0,5ng) (3 independent experiments; *n* = 46, *n* = 46 and *n* = 46), SB1-MO *tulp3* (0,5ng) (*n* = 43, *n* = 46 and *n* = 46), SB1-MO *tulp3* (0,5ng) + *HTULP3* mRNA (5pg) (*n* = 46, *n* = 46 and *n* = 46) or Co-MO (2ng) (3 independent experiments; *n* = 42, *n* = 39 and *n* = 34), SB2-MO *tulp3* (2ng) (*n* = 42, *n* = 40 and *n* = 36), SB2-MO *tulp3* (2ng) + *HTULP3* mRNA (5pg) (*n* = 38, *n* = 40 and *n* = 36). **(O)** Quantification of altered heart looping in 2dpf zebrafish embryos injected with Co-MO (2ng) (3 independent experiments; *n* = 41, *n* = 70 and *n* = 50 analysed embryos, respectively), TB-MO *tulp3* (2ng) (*n* = 43, *n* = 79 and *n* = 71), TB-MO *tulp3* (2ng) + *HTULP3* mRNA (5pg) (*n* = 56, *n* = 70 and *n* = 55) or Co-MO (0,5ng) (3 independent experiments; *n* = 46, *n* = 46 and *n* = 46), SB1-MO *tulp3* (0,5ng) (*n* = 43, *n* = 35 and *n* = 46), SB1-MO *tulp3* (0,5ng) + *HTULP3* mRNA (5pg) (*n* = 46, *n* = 46 and *n* = 46) or Co-MO (2ng) (3 independent experiments; *n* = 42, *n* = 39 and *n* = 34), SB2-MO *tulp3* (2ng) (*n* = 41, *n* = 39 and *n* = 40), SB2-MO *tulp3* (2ng) + *HTULP3* mRNA (5pg) (*n* = 39, *n* = 40 and *n* = 36); total number of embryos used for analyses are shown above respective bar. Unprocessed gel images **(B** and **C**, respectively**)** are presented in Suppl. Figure 7.

We used zebrafish as a vertebrate model organism to study a potential ciliary role of Tulp3 during embryonic development. While *tulp3* expression by whole-mount in situ hybridization (WISH) was not present, our previous analysis by semi-quantitative RT-PCR did identify *tulp3* expression during zebrafish embryogenesis^30^. We performed a Morpholino (MO)-based knockdown approach by using a translation-blocking MO (TB-MO) and two different splicing-blocking MOs (SB-MO) targeting the initiation codon of zebrafish *tulp3* mRNA and different exon-intron boundaries of zebrafish *tulp3* pre-mRNA, respectively (Fig. 1A-C). Embryos injected with either TB-MO *tulp3*, SB1-MO *tulp3* or SB2-MO *tulp3* revealed well-known ciliopathy-associated phenotypes such as pronephric cyst formation, otolith deposition defects, varying degrees of ventral body curvature and defective left-right (LR) asymmetry (analysed by the position of the heart looping) at 2 days post-fertilization (dpf) (Fig. 1D-O). None of these phenotypes were observed in embryos injected with a Control-MO. Co-injection of human *TULP3* mRNA and either TB-MO *tulp3*, SB1-MO *tulp3* or SB2-MO *tulp3* significantly prevented the observed phenotypes of Tulp3 morphant embryos, thus confirming MO specificity. Taken together, these results indicate a specific function for Tulp3 in cilia formation and/or function in zebrafish embryogenesis.

### Tulp3 knockout zebrafish present with ciliopathy-associated phenotypes during embryogenesis and adulthood

**Fig. 2:**
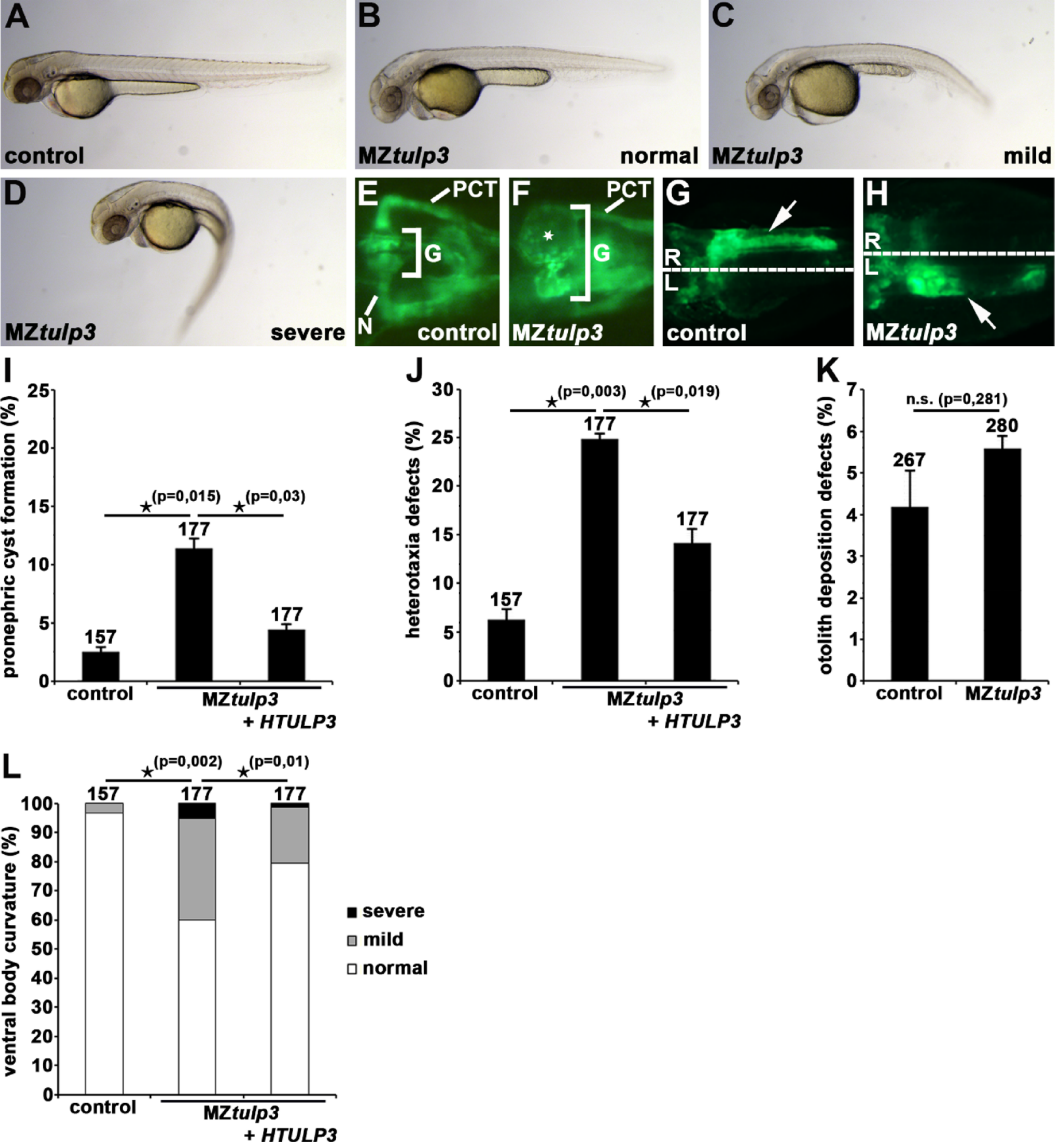
Tulp3 knockout analyses of cilia-related phenotypes during zebrafish embryogenesis.** (A-D)** Bright-field images of MZ*tulp3* embryos **(B-D)** and respective control embryo **(A)** at 2dpf. In comparison to the control embryos, MZ*tulp3* embryos display different degrees of ventral body curvature. **(E–K)** Knockout of Tulp3 leads to pronephric cyst formation (white star in **(F)**) and altered positioning of the exocrine pancreas (white arrow in **(H)**) at 2dpf as shown in a dorsal view with anterior to the left of a MZ*tulp3; li1Tg* embryo, respectively, in comparison to control embryos **(E**,** G)**; expression of EGFP fluorescence labels glomerulus (G), neck (N), proximal convoluted tubule (PCT) and exocrine pancreas. **(I)** Quantification of pronephric cyst formation in control embryos (3 independent experiments; *n* = 39, *n* = 59 and *n* = 59 analysed embryos, respectively), MZ*tulp3* embryos (*n* = 59, *n* = 59 and *n* = 59) and MZ*tulp3* embryos + *HTULP3* (*n* = 59, *n* = 59 and *n* = 59) at 2dpf. **(J)** Quantification of altered positioning of the exocrine pancreas in control embryos (3 independent experiments; *n* = 39, *n* = 59 and *n* = 59 analysed embryos, respectively), MZ*tulp3* embryos (*n* = 59, *n* = 59 and *n* = 59) and MZ*tulp3* embryos + *HTULP3* (*n* = 59, *n* = 59 and *n* = 59) at 2dpf. **(K)** Quantification of otolith deposition defects in control embryos (3 independent experiments; *n* = 101, *n* = 83 and *n* = 83 analysed embryos, respectively) and MZ*tulp3* embryos (*n* = 76, *n* = 92 and *n* = 112) at 2dpf. **(L)** Quantification of ventral body curvature in control embryos (3 independent experiments; *n* = 39, *n* = 59 and *n* = 59 analysed embryos, respectively), MZ*tulp3* embryos (*n* = 59, *n* = 59 and *n* = 59) and MZ*tulp3* embryos + *HTULP3* (*n* = 59, *n* = 59 and *n* = 59) at 2dpf; total number of embryos used for analyses are shown above respective bar.

**Fig.3:**
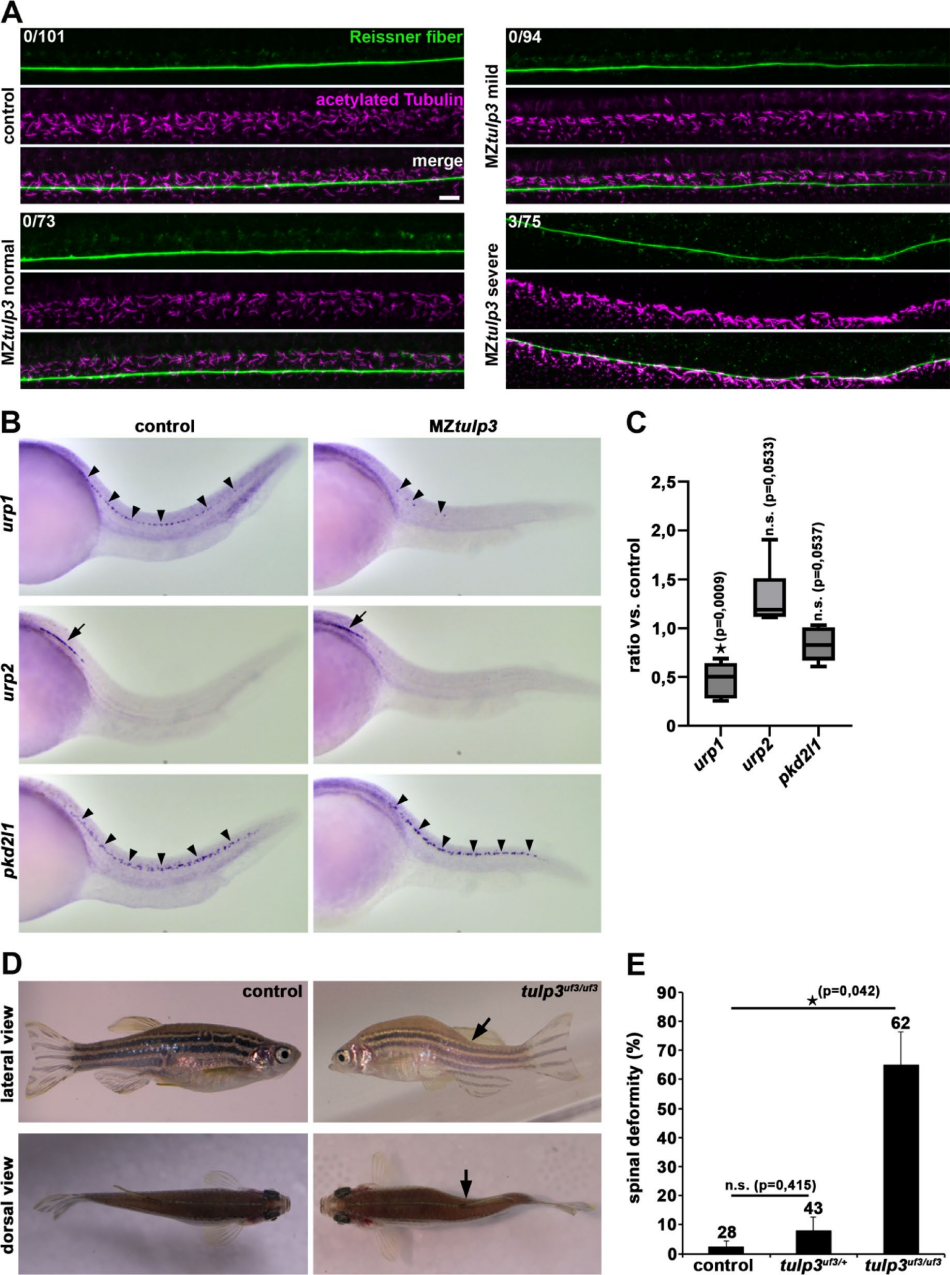
Tulp3 knockout results in decrease of *urp1* expression and scoliosis during zebrafish development. (A) Representative confocal images of MZ*tulp3* embryos (without curvature phenotype (normal), mild and severe ventral body curvature) and respective control embryos at 2dpf immunostained with anti-RF and anti-acetylated Tubulin as a ciliary marker. Loss of Tulp3 results in no obvious defects in RF formation compared to the control. Numbers represent embryos displaying RF disorganization and embryos that have been analysed in total from 3 independent experiments (control: *n* = 33, *n* = 42 and *n* = 26; MZ*tulp3* (normal): *n* = 11, *n* = 38 and *n* = 24; MZ*tulp3* (mild): *n* = 30, *n* = 28 and *n* = 36; MZ*tulp3* (severe): *n* = 22, *n* = 28 and *n* = 25). Scale bar: 10 μm. **(B)** WISH analysis reveals reduced *urp1* expression in ventral CSF-cNs (black arrowheads) of MZ*tulp3* embryos (presenting with a ventral curvature phenotype) compared to the control at 28hpf. Expression levels of *urp2* (black arrow) and *pkd2l1* (black arrowheads; serves as a control as its expression in CSF-cNs has been shown to be unaffected in ciliary mutants^43,48)^ are comparable between MZ*tulp3* and control embryos at 28hpf. **(C)** qPCR analysis reveals unaltered expression of *urp2* and *pkd2l1* while *urp1* expression was significantly reduced in MZ*tulp3* embryos compared to the respective control at 2dpf. **(D**,** E)** Representative images (lateral and dorsal views) and quantification of adult (18 months) *tulp3* mutant zebrafish displaying scoliosis phenotypes (black arrows) in comparison to the respective control analysed from 3 independent breedings (control: *n* = 15, *n* = 13 and *n* = 0; heterozygous *tulp3* mutant: *n* = 29, *n* = 6 and *n* = 8; homozygous *tulp3*: *n* = 15, *n* = 40 and *n* = 7); total number of adult fish used for analyses are shown above respective bar.

We previously analysed and described a CRISPR/Cas9-induced *tulp3* zebrafish knockout (*tulp3*^*uf3/*uf3^) presenting with severe liver and kidney phenotypes in adulthood^30^. We now focused on the effects of Tulp3 depletion on potential ciliopathy-associated phenotypes during zebrafish embryogenesis. Our analysis identified statistically significant pronephric cyst formation, defective LR-asymmetry (analysed by the position of the pancreas) and varying degrees of ventral body curvature in MZ *tulp3* knockout embryos at 2dpf; but we did not observe a statistical difference in otolith deposition (Fig. 2A-L). Injection of human *TULP3* mRNA into one-cell stage MZ*tulp3* embryos significantly prevented the observed phenotypes, confirming Tulp3 knockout specificity. Recent studies in zebrafish revealed that proper body axis morphogenesis relies on cilia-dependent formation of the RF, an acellular and filamentous structure that is present in the CSF^42,43^. In addition, Urp1 and Urp2, neuropeptides that are expressed in CSF-cNs, play a crucial role in signalling downstream of the RF in zebrafish^43–49^. Hence, we performed double whole-mount immunostainings on 2dpf old MZ*tulp3* embryos presenting with either a straight body axis or with a curvature phenotype, and respective control embryos using antibodies for RF and for the ciliary marker acetylated Tubulin. Our results revealed no obvious defects in RF formation in MZ*tulp3* embryos compared to the control at 2dpf (Fig. 3A). However, expression analyses of *urp* genes by WISH and quantitative real-time PCR (qPCR) studies revealed significantly reduced expression of *urp1* in MZ*tulp3* embryos compared to the control at 28 h post-fertilization (hpf) and 2dpf, respectively, while *urp2* expression was not significantly affected (Fig. 3B, C). In addition, analyses of adult *tulp3*^*uf3/*uf3^ zebrafish revealed ciliopathy-related spinal deformity compared to the respective heterozygous *tulp3* knockout or control siblings (Fig. 3D, E). In summary, these results indicate a role of Tulp3 in ciliogenesis, and most Tulp3 knockout data is in line with our Tulp3 knockdown data.

### Tulp3 deficiency results in defective ciliogenesis and disrupted cilia-related signalling

**Fig. 4:**
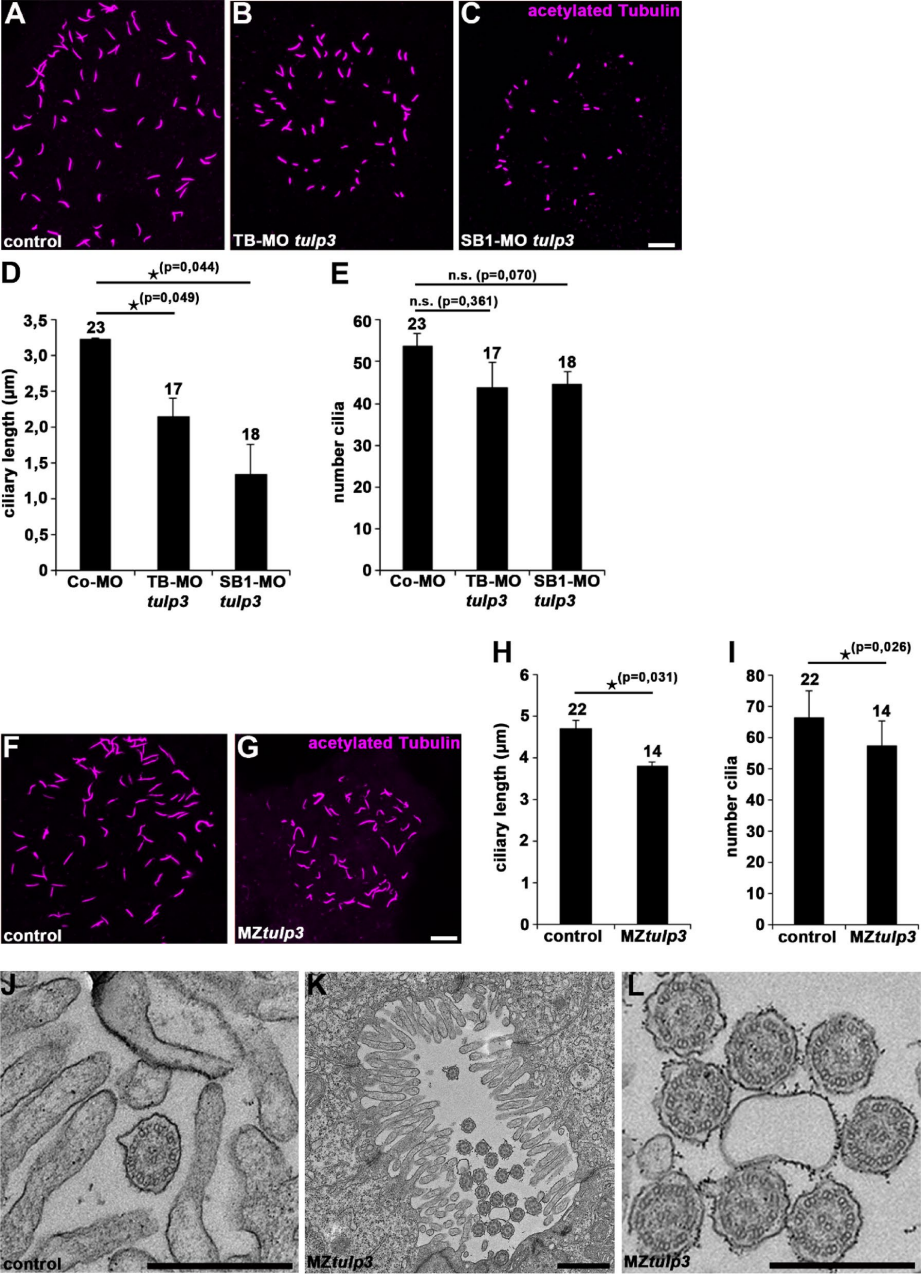
Tulp3 deficiency results in defective cilia formation and function in zebrafish.** (A-C)** Representative confocal images of the Kupffer’s vesicle of Co-MO (2ng) **(A)**, TB-MO *tulp3* (2ng) **(B)** or SB1-MO *tulp3* (0,5ng) **(C)** injected embryos at 8 somites immunostained with anti-acetylated Tubulin as a ciliary marker. Scale bar: 10 μm. **(D**,** E)** Quantification of the ciliary length **(D)** and cilia number **(E)** in the Kupffer’s vesicle of Co-MO (3 independent experiments; *n* = 9, *n* = 7 and *n* = 7 analysed embryos, respectively), TB-MO *tulp3* (*n* = 6, *n* = 4 and *n* = 7) or SB1-MO *tulp3* (*n* = 4, *n* = 6 and *n* = 8) injected embryos at 8 somites; total number of embryos used for analyses are shown above respective bar. **(F**,** G)** Representative confocal images of the Kupffer’s vesicle of MZ*tulp3* embryo **(G)** and respective control embryo **(F)** at 8 somites immunostained with anti-acetylated Tubulin. Scale bar: 10 μm. **(H**,** I)** Quantification of the ciliary length **(H)** and cilia number **(I)** in the Kupffer’s vesicle of control embryos (3 independent experiments; *n* = 7, *n* = 7 and *n* = 8 analysed embryos, respectively) and MZ*tulp3* embryos (*n* = 6, *n* = 4 and *n* = 4) at 8 somites; total number of embryos used for analyses are shown above respective bar. **(J-L)** TEM of pronephric tubule microvilli and motile cilia of control embryos **(J)** and MZ*tulp3* **(K**,** L)** embryos (with severe ventral body curvature) at 5dpf. Scale bars: 500 nm **(J**,** L)**; 1 μm **(K)**.

**Fig. 5:**
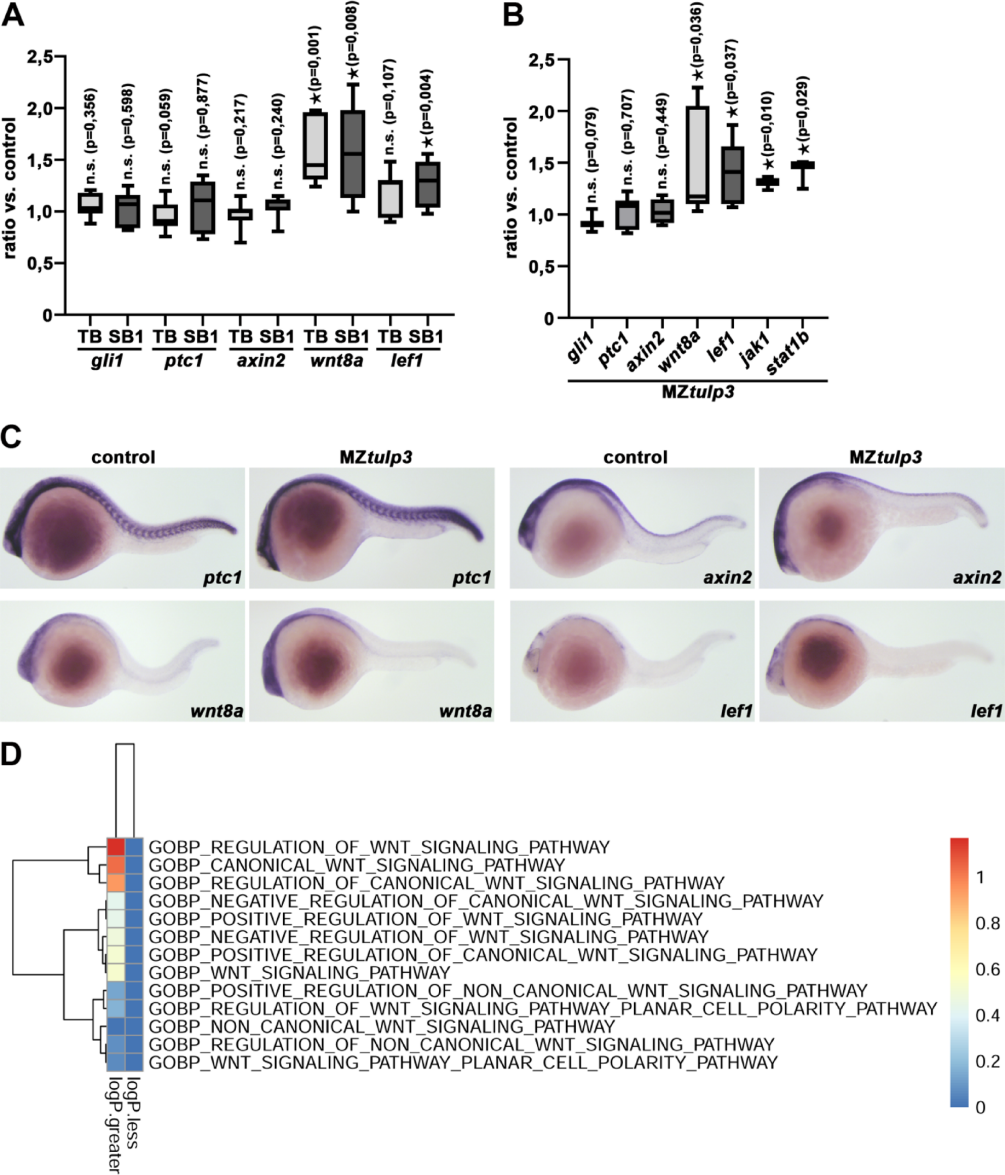
Tulp3 deficiency results in the upregulation of cilia-dependent signalling pathway components in zebrafish. **(A)** qPCR analysis reveals unaltered expression of Hh signalling components (*gli1*, *ptc1*) and Wnt signalling component *axin2* while Wnt signalling components *wnt8a* and *lef1* were upregulated upon TB/SB1-MO *tulp3* mediated knockdown compared to the respective control at 1dpf. **(B)** qPCR analysis reveals unaltered expression of Hh signalling components (*gli1*, *ptc1*) and Wnt signalling component *axin2* while Wnt signalling components *wnt8a* and *lef1* or Jak/Stat signalling components *jak1* and *stat1b* were upregulated in MZ*tulp3* embryos compared to the respective control at 1dpf. **(C)** WISH analyses indicate unaltered expression of Hh signalling component *ptc1* and Wnt signalling component *axin2* while Wnt signalling components *wnt8a* and *lef1* were upregulated in MZ*tulp3* embryos compared to the respective control at 1dpf. **(D)** Heatmap of Wnt signalling pathway associated gene ontology (GO)-terms (biological process, BP) of MZ*tulp3* embryos compared to control embryos at 2dpf. Columns represent up- or downregulated processes in MZ*tulp3* mutant embryos compared to controls.

Previous data demonstrated reduced ciliary length in various Tulp3 deficient or depleted cell types^20^. In order to analyse cilia formation in Tulp3 morphant and mutant zebrafish embryos we performed acetylated Tubulin immunostaining to visualize the cilia in the Kupffer’s vesicle (the organ of laterality in zebrafish) at the stage of 8 somites. Quantification revealed statistically significant reduced ciliary length in Tulp3 deficient and depleted embryos compared to the respective controls (Fig. 4A-H). In addition, the number of cilia was significantly reduced in MZ*tulp3* embryos compared to the control (Fig. 4I). Transmission electron microscopy (TEM) studies of motile cilia in the pronephric tubules revealed normal ciliary architecture with nine outer doublet microtubules surrounding a central pair of singlet microtubules in control as well as MZ*tulp3* embryos (Fig. 4J-L). We also performed qPCR and WISH analyses to analyse cilia-dependent signalling pathways in Tulp3 deficient and depleted embryos at 1dpf. While Hh signalling was unaffected, we observed an upregulation of Wnt and Jak/Stat signalling pathway components in Tulp3 morphant and mutant embryos compared to the respective controls (Fig. 5A-C). In addition, we performed RNA sequencing (RNA-Seq) analyses on MZ*tulp3* embryos in comparison to the control at 2dpf. Our results revealed upregulation of Wnt signalling associated pathways in MZ*tulp3* embryos, while Hh signalling was unaffected (Fig. 5D, Suppl. Figures 1 and 2).

### Loss of Tulp3 leads to upregulation of genes related to liver fibrosis

**Fig. 6:**
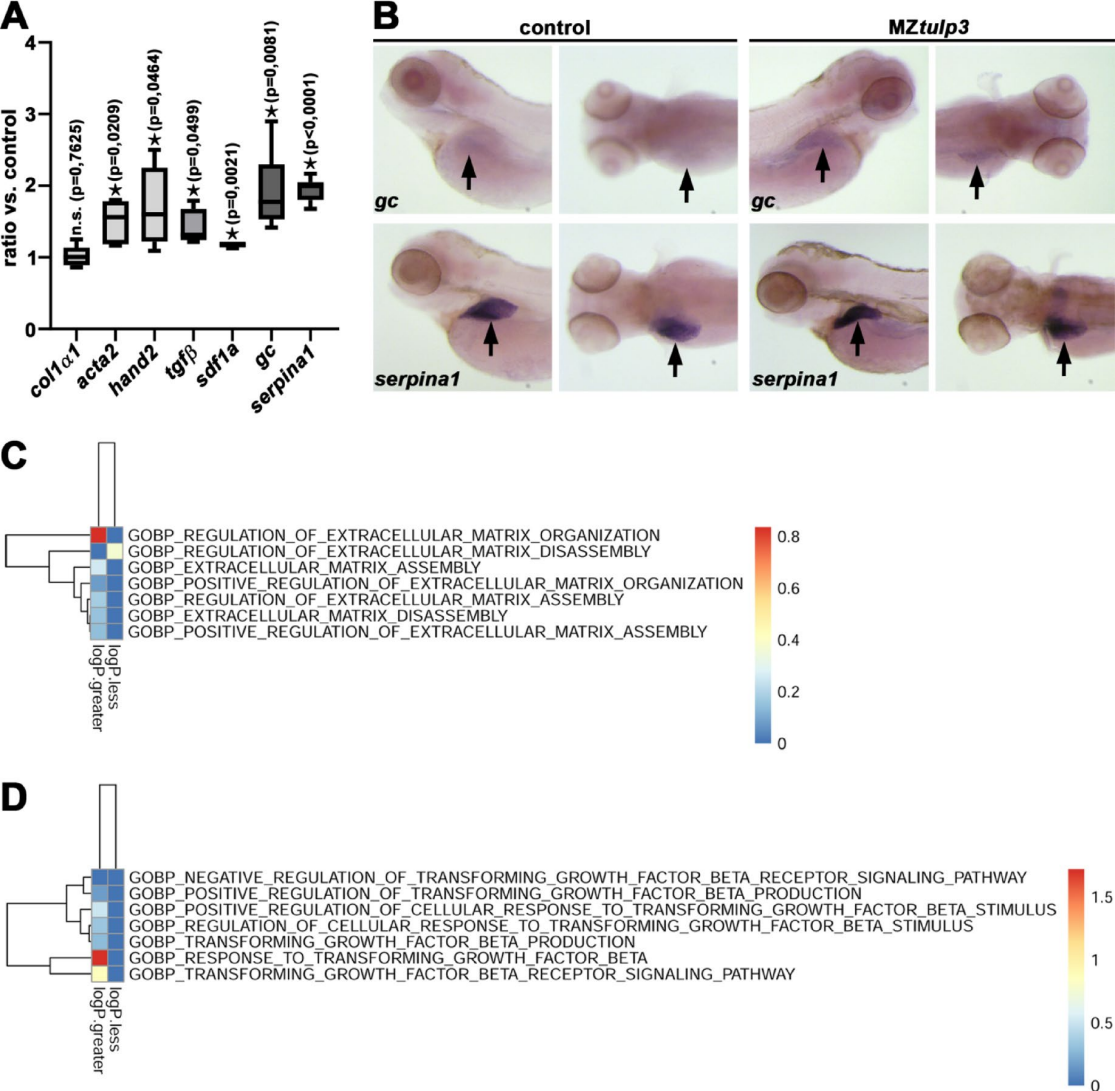
Loss of Tulp3 results in the upregulation of genes related to fibrosis during zebrafish embryogenesis.** (A)** qPCR analysis reveals unaltered expression of *col1α1* while genes related to liver-fibrosis (*acta2*, *hand2*), inflammatory/damage (*tgfβ*, *sdf1a*) and liver function (*gc*, *serpina1*) were significantly upregulated in MZ*tulp3* embryos compared to the respective control at 4dpf. **(B)** WISH analyses indicate upregulated expression of *gc* and *serpina1* in the liver (black arrow) in MZ*tulp3* embryos compared to the respective control at 4dpf (for each condition a lateral and dorsal view is shown). **(C**,** D)** Heatmaps of ECM organization **(C)** and Tgfβ signalling pathway **(D)** indicate an increase in related biological processes (GOBP) in MZ*tulp3* mutant compared to control embryos at 2dpf. Shown are respective up- or downregulated BPs.

Our previous data demonstrated that *tulp3*^*uf3/*uf3^ knockout zebrafish developed liver fibrosis during adulthood^30^. We now aimed to study whether signs of liver fibrosis are already detectable during zebrafish embryogenesis upon loss of Tulp3. Therefore, we performed qPCR and WISH to analyse the expression levels of genes related to liver fibrosis, inflammatory/damage and liver function in MZ*tulp3* embryos compared to the control at 4dpf^50^. Our results revealed significant upregulation of the fibrotic (*hand2* and *acta2*), inflammatory/damage (*tgfß* and *sdf1a*) and function (*gc* and *serpina1*) related genes while expression of liver fibrosis-related gene *col1α1* was not affected (Fig. 6A, B). In addition, our RNA-Seq analyses indicated an increase in fibrosis-related extracellular matrix (ECM) organization and upregulation of Tgfβ-signalling pathway in MZ*tulp3* mutant embryos compared to the control at 2dpf (Fig. 6C, D and Suppl. Figures 3 and 4).

## Discussion

In humans, bi-allelic deleterious variants in *TULP3* are the cause of a progressive degenerative disease affecting the liver, kidney and heart tissue^30,31^. Disease onset, presenting with first signs of liver disease, mostly varies from early childhood to the mid-twenties. The progressive course of TULP3-dependent fibrocystic liver and kidney disease emphasizes that, for the identification of potential targets for therapeutic actions to circumvent or delay organ dysfunction, a detailed understanding of the disease mechanism is very important.

Previous mouse models showed that the loss of TULP3 function results in neural tube closure defects, craniofacial abnormalities, polydactyly and embryonic lethality^26–29^. In contrast to *Tulp3* knockout mice, zygotic *tulp3* knockout zebrafish survive into adulthood even though they developed fibrocystic liver and kidney disease phenotypes^30^. In addition, we now document a scoliosis phenotype in zygotic adult *tulp3* mutants. To further study the progressive character of the disease, we have examined Tulp3 loss of function phenotypes during embryonic stages in zebrafish by using morpholino (MO)-mediated knockdown and CRISPR/Cas9-mediated knockout approaches. Notably, inconsistencies of phenotypes observed either by MO-mediated knockdown or gene-editing-based knockout in zebrafish have been documented by recent reports^51^. A subsequent study identified the activation of a genetic compensation response in mutants but not in morphants^52^. Following up on these observations, a further study reported that genetic compensation is triggered by the generation of unstable mRNAs^53^. Of note, compensation mechanisms and their investigation are likely to be complex and far from being completely understood^54^. Hence, it is widely accepted that gene functional analyses in zebrafish are ideally based on two or more different approaches, complementing knockout strategies with additional genetic tools^55^. We strongly agree with this view and therefore used the MO-mediated knockdown as well as knockout approach to study Tulp3 function in zebrafish embryogenesis. By the majority (except otolith deposition defects), we observe similar phenotypes with differing penetrance/severity between the morphant and mutant data, confirming specificity of our experimental data. Our analyses revealed less severe phenotypes in the MZ*tulp3* mutant embryos compared to *tulp3* morphants. Therefore, we addressed a possible genetic compensation in MZ*tulp3* mutant embryos by analysing gene expression of tulp3 related family members and known interactors by RNA-Seq analysis. However, our results revealed no significant upregulation of selected compensatory candidate genes (Suppl. Figure 5). We cannot exclude that other possible genetic compensation mechanisms and/or transcriptional adaptation might be active in MZ*tulp3* mutants and thus is a matter of future investigations. In addition, we cannot exclude the possibility that the MOs induce an off-target effect that enhances the on-target phenotypes.

We here report that Tulp3 deficiency results in various well-known cilia-associated phenotypes. Previous data revealed a reduction in ciliary length in various *Tulp3* knockout cells while the percentage of cilia was normal^20^. In line with these findings, our results revealed a significant reduction in ciliary length in Tulp3 deficient embryos, but also showed that the number of cilia is significantly reduced in *tulp3* knockout embryos. It is noteworthy that our TEM analyses identified an unaffected ciliary architecture in *tulp3* knockout embryos. We also demonstrate that Tulp3 deficiency results in ventral body curvature defects, a phenotype that is linked to dysfunctional cilia. Recent studies identified the importance of cilia-driven cerebrospinal fluid (CSF) flow in transmitting adrenergic signals to CSF-contacting neurons that subsequently promote the synthesis and secretion of Urp1 and Urp2. Subsequently, Urp1/Urp2 bind to and activate their receptor Uts2r3 on dorsal somitic muscles to promote axis straightening. Thus, signals from CSF finally direct dorsal muscle fiber contraction and control proper body axis straightening during early development^42,44,45,48^. Our results revealed significant downregulation of *urp1* in MZ*tulp3*. How Tulp3 deficiency affects *urp1* expression still remains unknown. While we detect perturbation in *urp1* expression and axial curvature phenotypes, our analyses of immunostained embryos followed by confocal microscopy revealed no obvious RF formation defects. Despite the formation of a continuous RF, it is still possible that the CSF signalling is affected in MZ*tulp3* embryos. Notably, a recent study demonstrated a scoliotic model with motile cilia and CSF flow defects but intact RF formation and unaffected *urp* gene expression showing that the curvature onset occurs independently of RF loss in this model^56^. In addition, neuro-inflammation has been described in a small subset of zebrafish IS models^57,58^. Conclusively, recent studies identified a variety of factors that lead to scoliosis but several aspects in respect to the regulatory mechanisms underlying spinal deformity still remain unknown and need to be addressed in future studies. Recent analyses of cilia-dependent signalling pathways in *Tulp3* mutant mice identified TULP3 as a negative regulator of the HH signalling pathway^27–29^. In MZ*tulp3* mutant zebrafish embryos we did not detect defects in Hh target genes *gli1* or *ptc1* and RNA-Seq analyses showed no dysregulation in key target genes of Hh signalling. Notably, we observed a significant increase in the ciliary TZ protein encoding gene *ttc23* in MZ*tulp3* mutant embryos that will need further validation. In addition, TTC23 has been described to participate in Hh signalling^59^. In Tulp3 morphant and MZ*tulp3* mutant embryos we found a significant increase in expression of profibrotic Wnt and Jak/Stat signalling components. Furthermore, our RNA-Seq analysis underlined the upregulation of Wnt associated signalling pathways in MZ*tulp3* mutant embryos. Notably, these in vivo results are in perfect line with our previous in vitro data of a *TULP3* variant carrying individual that demonstrated unaltered HH signalling but an upregulation of WNT and JAK/STAT signalling components^30^. Thus, the contradictory results indicate that TULP3 has different functions in the context of cilia-related signalling pathways that have been analysed in human and the model organisms zebrafish and mouse. Dysregulation of WNT and JAK/STAT signalling have been linked to fibrosis of major organs and cystic kidney disease, phenotypes that we observed in adult homozygous *tulp3* mutants^30,60–62^. We also examined if signs of liver fibrosis are already detectable in Tulp3 deficient embryos. Indeed, we observed that genes related to liver fibrosis are upregulated in MZ*tulp3* embryos, which is in line with our observation of liver fibrosis in adult homozygous *tulp3* mutants^30^. Noteworthy, our RNA-Seq analyses further indicated an increase of various oncogenic and profibrotic cell processes, comparable to results that we previously reported in a *TULP3*-affected individual (Suppl. Figure 6)^30^.

Conclusively, we here present cilia-related phenotypes during zebrafish embryogenesis that are caused by deficiency of Tulp3 function. These results indicate a progressive disease character not only in humans but also in zebrafish. Therefore, our data demonstrates that the Tulp3 loss of function zebrafish model represents a suitable disease model to study progressive fibrocystic liver and kidney disease.

## Materials and methods

### Zebrafish lines and embryo maintenance

The fish used in this study were maintained at the Zebrafish Facility of the Medical Center of the University of Freiburg. All animal work has been conducted according to relevant national and international guidelines. The study was approved by the Institutional Animal Care of the Medical Center of the University of Freiburg and the Regional Council Freiburg (permit ID G-16/89). All methods were carried out in accordance with ARRIVE guidelines. Zebrafish were maintained and the embryos were staged as previously described^63^. The following strains were used: AB/TL wildtype (WT), *li1Tg* (https://zfin.org/ZDB-ALT-071127-1)^64^ and *tulp3*^*uf3/uf3*^; *li1Tg (AB/TL)* (https://zfin.org/ZDB-FISH-230405-2)^30^.

### mRNA and morpholino (MO) injections

For synthesis of mRNA, full-length human Tulp3 was cloned into pCS2 + MxN, linearized with NsiI, and transcribed using SP6 mMessage mMachine Kit (Ambion). mRNA and Morpholino oligonucleotide (MO) injections were performed as described^65^. To attenuate possible off target effects, a p53 MO was co-injected 1.5-fold to the other MOs used^66^. The following translation/splicing-blocking (TB/SB) antisense MOs (Gene Tools) were used for zebrafish: TB-MO *tulp3* 5′-CTCTTCACCGTCTCCATGTCGAG-3′, SB1-MO *tulp3* 5′-TTGTTCTGTGTGTGTCTTACCGCGT-3′, SB2-MO *tulp3* 5’-TGTGCAGTTGGACTGACGTAGCATA-3’, TB-MO *p53* 5’-GCGCCATTGCTTTGCAAGAATTG-3’^66^ and a Standard Control (Co)-MO. The Co-MO is thought to have no target and only very little biological activity (https://www.gene-tools.com/custom_morpholinos_controls_endmodifications).

### Reverse-transcription polymerase chain reaction (RT-PCR) analysis

Semiquantitative RT-PCR was performed to determine expression of zebrafish *tulp3*. Total RNA from entire 1dpf old zebrafish embryos was extracted with the RNeasy Kit (Qiagen), followed by complementary DNA (cDNA) synthesis with the ProtoScript First Strand cDNA Synthesis Kit (Promega). Analysis of zebrafish ef1α was used as a loading control. The following primers were used for PCR analysis: tulp3 (forward-1: 5ʹ-TCTGCTGGAGCAGAAGCAG-3ʹ, reverse-1: 5ʹ-GTGGATTTAGTGCTGGATGCAGAC-3ʹ; for analysis of SB1-MO *tulp3* efficiency); tulp3 (forward-2: 5ʹ-CATCCAGCACTAAATCCACTAC-3ʹ, reverse-2: 5ʹ-GTAAGACTGTGTGTCATCGTTC-3ʹ; for analysis of SB2-MO *tulp3* efficiency); ef1α (forward: 5ʹ-ATCTACAAATGCGGTGGAAT-3ʹ, reverse: 5ʹ-ATACCAGCCTCAAACTCACC-3ʹ).

### Synthesis of antisense RNA and in situ analysis

Zebrafish *urp1*, *urp2*,* pkd2l1* and *lef1* probes were amplified from zebrafish cDNA with primers (urp1, forward: 5ʹ-ACATTCTGGCTGTGGTTTG-3ʹ, reverse: 5ʹ-TGTATGGGGAAAACAAAGG-3ʹ)^67^; (urp2, forward: 5ʹ-CGACGCGAGCATTAGATGAA-3ʹ, reverse: 5ʹ-TGTTGGTTTTCTTGGTTGACG-3ʹ); (pkd2l1, forward: 5ʹ-GTGACTGTTTCGATGTGTAC-3ʹ, reverse: 5ʹ-ACGATCTCACAGCCGATGAT-3ʹ); (lef1, forward: 5ʹ-CTGGACCCCACGCCACAGGAAT-3ʹ, reverse: 5ʹ-GGCCTGTAGCTGCTGTCTTTGCTT-3ʹ). Zebrafish *gc* and *serpina1* probes were amplified from zebrafish cDNA with primers that have been used previously in quantitative real-time PCR analyses^50^. Amplification products were cloned into TOPO (Invitrogen), followed by sequence verification and linearization with corresponding restriction enzymes for synthesis of antisense RNA. We used already described pBS-ptc1, pCRII-axin2 and pGEMT-wnt8a for synthesis of antisense RNA^68–70^. Whole-mount in situ hybridization (WISH) analysis using Digoxigenin-labelled probes was performed as described using NBT (blue) (Roche) as substrate^71^.

### Quantitative real‑time PCR (qPCR)

qPCR was performed as previously described^72^. For the expression analysis of *urp1*, *urp2* and *pkd2l1*, total RNA was obtained from 30 maternal zygotic (MZ) *tulp3* mutant embryos presenting with ventral body curvature or from respective control embryos at 2dpf using the RNeasy Kit (Qiagen). The following primers were used for qPCR analysis: ef1α (forward: 5ʹ-TGCCAACTTCAACGCTCAGGTC-3ʹ, reverse: 5ʹ-TCAGCAAACTTGCAGGCGATG-3ʹ); urp1 (forward: 5ʹ-ACATTCTGGCTGTGGTTTG-3ʹ, reverse: 5ʹ-GTCCGTCTTCAACCTCTGCTAC-3ʹ); urp2 (forward: 5ʹ-AGAGGAAACAGCAATGGACG-3ʹ, reverse: 5ʹ-TGTTGGTTTTCTTGGTTGACG-3ʹ); pkd2l1 (forward: 5ʹ-GTGACTGTTTCGATGTGTAC-3ʹ, reverse: 5ʹ-CTTGATAAAACCCTGCTCCG-3ʹ)^43^. For the analysis of Hh, Wnt and Jak/Stat signalling pathway components, total RNA was obtained from 30 Co-MO or TB/SB1-MO *tulp3* injected zebrafish embryos and 30 unbiasedly pooled MZ*tulp3* mutant or from respective control embryos, all at 1dpf using the RNeasy Kit (Qiagen). The following primers were used for qPCR of analysis: ef1α (forward: 5ʹ-TGCCAACTTCAACGCTCAGGTC-3ʹ, reverse: 5ʹ-TCAGCAAACTTGCAGGCGATG-3ʹ); gli1 (forward: 5ʹ-TCAGACGTCCTCTCGCCTTA-3ʹ, reverse: 5ʹ-AGCTCATGTCTCCGATTGCC-3ʹ); ptc1 (forward: 5ʹ-GGGTCCTGAATGGACTGGTG-3ʹ, reverse: 5ʹ-CCGCTGGAGATACCTCAGGA-3ʹ); axin2 (forward: 5ʹ-ACCCTCGGACACTTCAAGGA-3ʹ, reverse: 5ʹ-GTGCAGTCATCCCAGACCTC-3ʹ); wnt8a (forward: 5ʹ-ATTCGTGGATGCGCTTGAGA-3ʹ, reverse: 5ʹ-TTACAGCCAAACGTCCAGCTT-3ʹ); lef1 (forward: 5ʹ-CAGACATTCCCAATTTCTATCC-3ʹ, reverse: 5ʹ-TGTGATGTGAGAACCAACC-3ʹ); jak1 (forward: 5ʹ-CCTGGAGGAGGGAAAGAGAC-3ʹ, reverse: 5ʹ-CAGGCTTTTGAAGTCGATCC-3ʹ); stat1b (forward: 5ʹ-CTCCAGGCACTTTCCTTCTG-3ʹ, reverse: 5ʹ-AATGGATCTTGGGTTCACCA-3ʹ). For the analysis of genes related to liver-fibrosis, total RNA was obtained from 30 MZ*tulp3* mutant or respective control embryos at 4dpf using the RNeasy Kit (Qiagen). For qPCR analysis we used the primers for ef1α and the primers that have been used previously^50^.

### Immunostaining

Zebrafish whole-mount immunofluorescence (IF) was performed as previously described^65^. The following antibodies were used for IF: anti-acetylated αTubulin (clone 6-11B-1; Sigma Aldrich; 1:500) and anti-Reissner fiber (kind gift from Stéphane Gobron; 1:200). Cy3 (1:1000) and Alexa-488 (1:1000) labelled secondary antibodies were purchased from Jackson Immunoresearch and Molecular Probes (Invitrogen), respectively.

### Microscopy and image acquisition

Embryos were analysed using a Leica MZ16F epifluorescent microscope. Images were obtained with a Leica DFC 450 C camera and processed with Leica Application Suite X (Version 1.4.5) (https://www.leica-microsystems.com/products/microscope-software/details/product/leica-las-x-ls/). Confocal images of whole-mount zebrafish immunostainings were generated with a Carl Zeiss LSM510 laser scanning microscope (ZEISS objectives: Achroplan NIR 40x/0.8 water-immersion). Confocal z-stacks were projected to one plane (maximum intensity projection). Confocal images of whole-mount zebrafish immunostainings were generated with a Carl Zeiss LSM510 laser scanning microscope. The transmission electron microscopy (TEM) procedure is described elsewhere^73^. All images were exported as TIFF files and imported into Adobe Photoshop Creative Suite 2 (Version 11) (https://www.adobe.com/de/products/photoshop.html) to arrange figures.

### RNA sequencing and analysis

RNA sequencing was performed by Novogene. Total RNA was isolated from MZ*tulp3* (presenting with severe ventral body curvature) and respective control embryos at 2dpf using the RNeasy Kit (Qiagen). The raw RNA sequencing files were processed with the nf-core/rnaseq workflow (v3.18.0) including pre-processing with TrimGalore! to ensure sufficient read quality by removing adapters and reads in low-quality segment regions^74^. Subsequently, the reads were 2-pass aligned using the STAR aligner and quantified with RSEM. The Danio rerio genome GRCz10 was used as reference. Normalization and differential expression analysis was done with the R/Bioconductor package DESeq2 (v1.42.1)^75–77^. Genes were considered significant with an adjusted p-value (FDR corrected, according to Benjamini-Hochberg) < 0.05.

### Gene set enrichment analysis

Gene set enrichment analysis (GSEA) of signalling pathways was performed as implemented in the R/Bioconductor package GAGE (Generally Applicable Gene-set Enrichment analysis), with signalling pathways from the Molecular Signatures Database (MSigDB)^78,79^. In particular the Gene ontology (GO) gene set Biological Processes (BP) (https://geneontology.org/docs/ontology-documentation/) and the MSigDB hallmark gene set collection were used^80^. Pathways were considered significant with an adjusted p-value (Benjamini-Hochberg) < 0.05. For better visualization, expression values of genes associated with specific GSEA terms were z-scaled.

### Statistical analysis and quantification

For each quantification, the number of independent experiments and the number of embryos is specified in the respective figure legend. Total numbers of embryos used for analysis are indicated in the respective bar chart unless otherwise stated. Data were analysed by Student’s *t*-test (2-sided, unpaired); error bars represent the standard error of the mean (SEM). Measurement and quantification of ciliary length is described elsewhere^81^. qPCR data were analysed with GraphPad Prism (Version 10.5.0) (https://www.graphpad.com/updates/prism-10-5-0-release-notes) and one sample *t*-test; error bars represent the SEM.

### Accession numbers

Corresponding GenBank accession number for human cDNA: Tulp3 (NM_003324.5) and zebrafish cDNA: tulp3 (XM_005164458.6).

## Supplementary Information

Below is the link to the electronic supplementary material.

Supplementary Material 1

## Data Availability

The RNA-Seq datasets generated and/or analysed during the current study are available in the GEO repository, https://www.ncbi.nlm.nih.gov/geo/query/acc.cgi?acc=GSE300126. All other datasets used and/or analysed during the current study are available from the corresponding authors on reasonable request.
