# Correction: Tulp3 deficiency results in ciliopathy phenotypes during zebrafish embryogenesis

**DOI:** 10.1038/s41598-025-31559-0

**Published:** 2026-01-12

**Authors:** Daniel Epting, John Devane, Ralf Mertes, Séverine Kayser, Martin Helmstädter, Patrick Metzger, Melanie Boerries, Carsten Bergmann, Elisabeth Ott

**Affiliations:** 1 Department of Medicine IV, Faculty of Medicine, Medical Center-University of Freiburg, University of Freiburg, Freiburg, Germany; 2 Institute of Medical Bioinformatics and Systems Medicine, Faculty of Medicine, Medical Center-University of Freiburg, University of Freiburg, Freiburg, Germany; 3 Faculty of Biology, University of Freiburg, Freiburg, Germany; 4 Faculty of Medicine, University of Freiburg, Freiburg, Germany; 5 German Cancer Consortium (DKTK), Partner site Freiburg, DKFZ and Medical Center-University of Freiburg, Freiburg, Germany; 6 Medizinische Genetik Mainz, Limbach Genetics, Mainz, Germany

Correction to: *Scientific Reports* 10.1038/s41598-025-16584-3, published online 12 September 2025

The original version of this Article contained an error in the Reference list, where Reference 31 was incorrectly given as Reference 50.

The correct Reference 31 is listed below:

Jafari Khamirani, H. et al. A pathogenic variant of TULP3 causes renal and hepatic fibrocystic disease. *Front. Genet.* **13**, 1021037. 10.3389/fgene.2022.1021037 (2022).

As a result, References have been renumbered accordingly.

The original Article has been corrected.

