# Supplementary material for: Tulp3 deficiency results in ciliopathy phenotypes during zebrafish embryogenesis
Source: Sci Rep. 2025 Sep 12;15:32435. doi: 10.1038/s41598-025-16584-3 (PMC12432116; doi:10.1038/s41598-025-16584-3)
Supplement: Supplementary file 1 — Supplementary Material 1 [file 41598_2025_16584_MOESM1_ESM.pdf]

# Supplementary Information

## Tulp3 deficiency results in ciliopathy phenotypes during zebrafish embryogenesis

**Daniel Epting, John Devane, Ralf Mertes, Séverine Kayser, Martin Helmstädter, Patrick Metzger, Melanie Boerries, Carsten Bergmann and Elisabeth Ott**

**Suppl. Fig. 1 Gene set enrichment analysis of Wnt signalling.**

**Suppl. Fig. 2 Differential gene expression associated with Hh signalling.**

**Suppl. Fig. 3 Gene set enrichment analysis of ECM-related processes.**

**Suppl. Fig. 4 Gene set enrichment analysis of Tgfβ signalling.**

**Suppl. Fig. 5 Gene expression heatmap of Tulp3 associated genes.**

**Suppl. Fig. 6 Analyses of oncogenic and fibrosis-related processes.**

**Suppl. Fig. 7 Respective uncropped and unprocessed gel images.**

**Supplementary Figure 1**

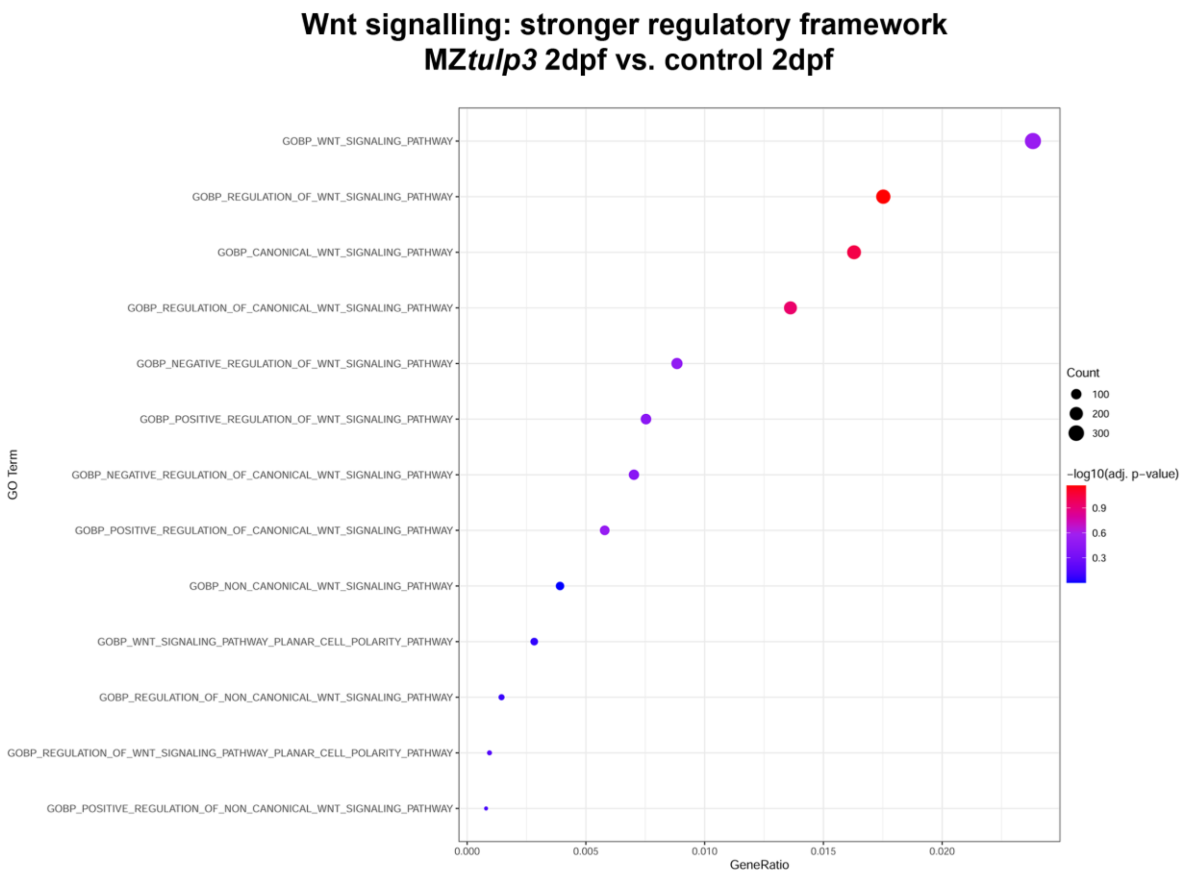

**Supplementary Figure 2**

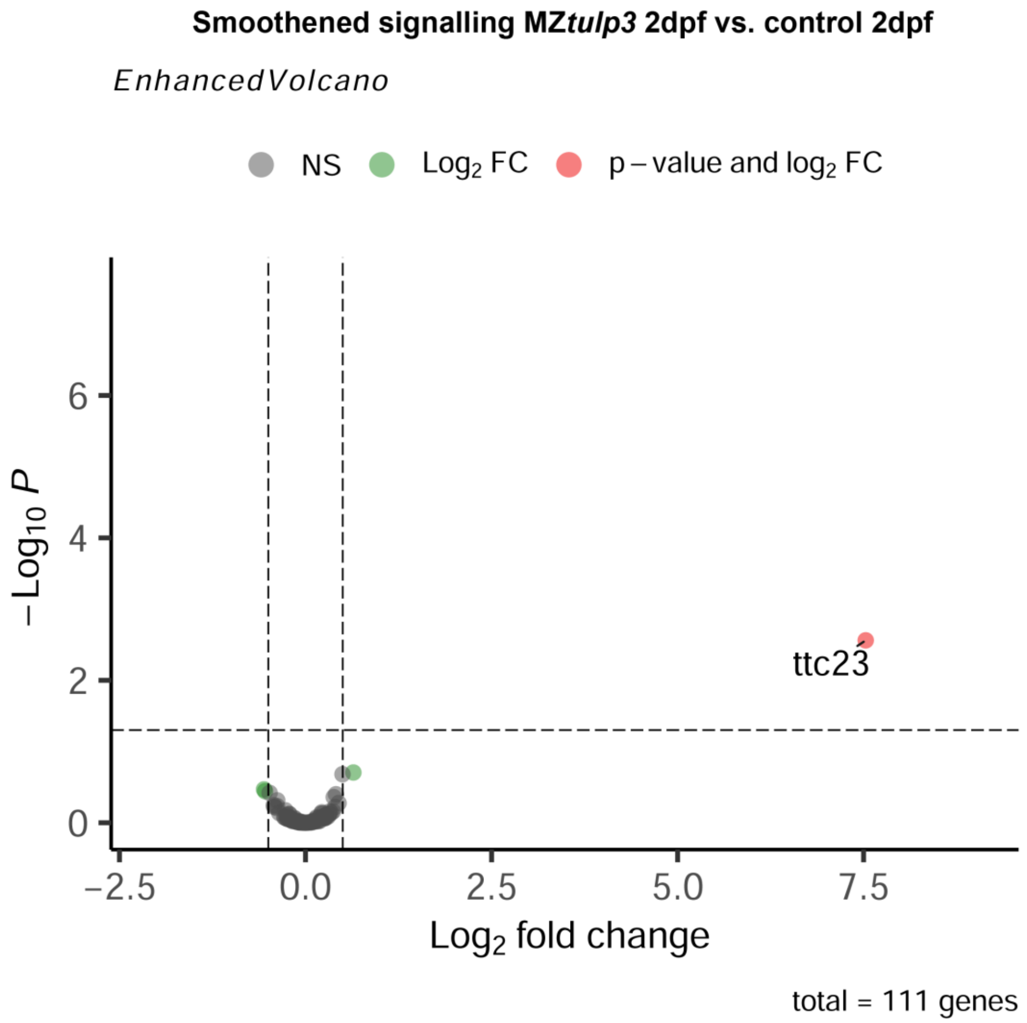

## Supplementary Figure 3

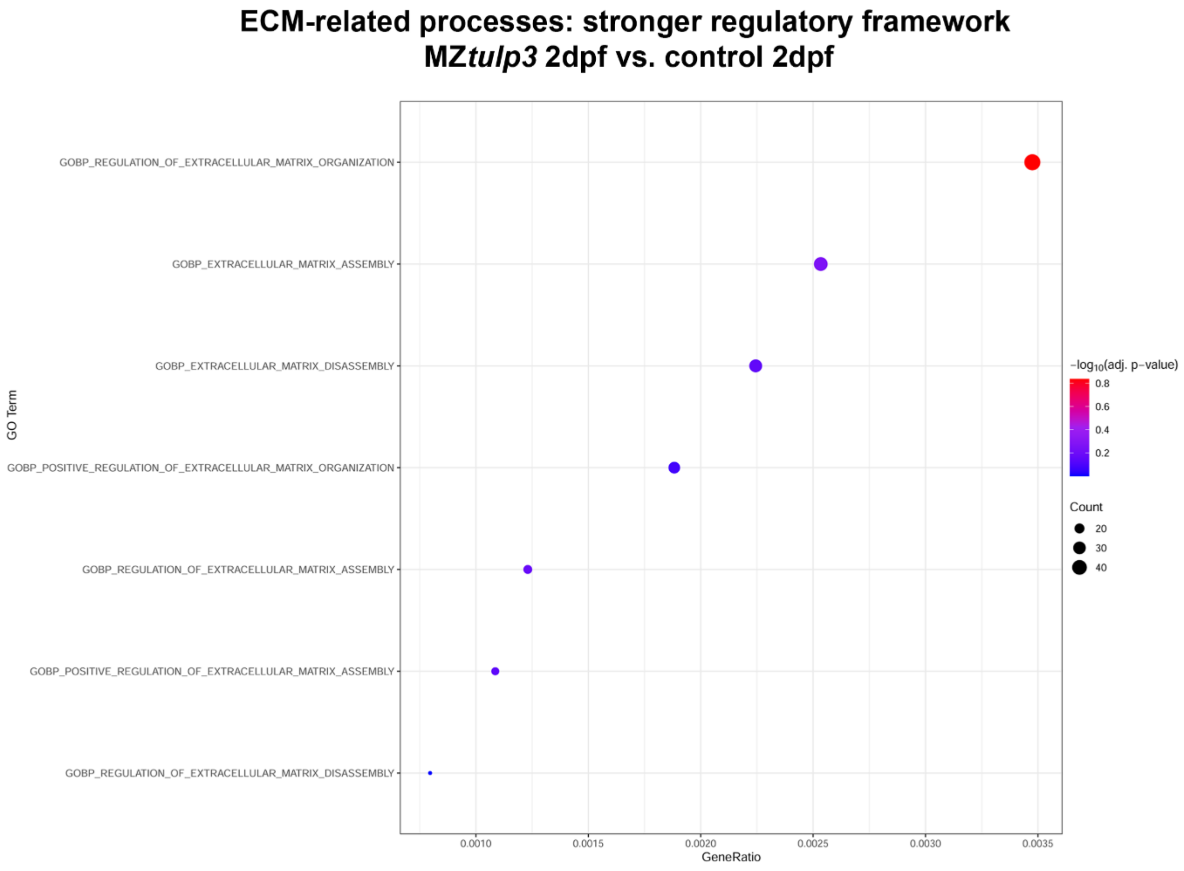

**Supplementary Figure 4**

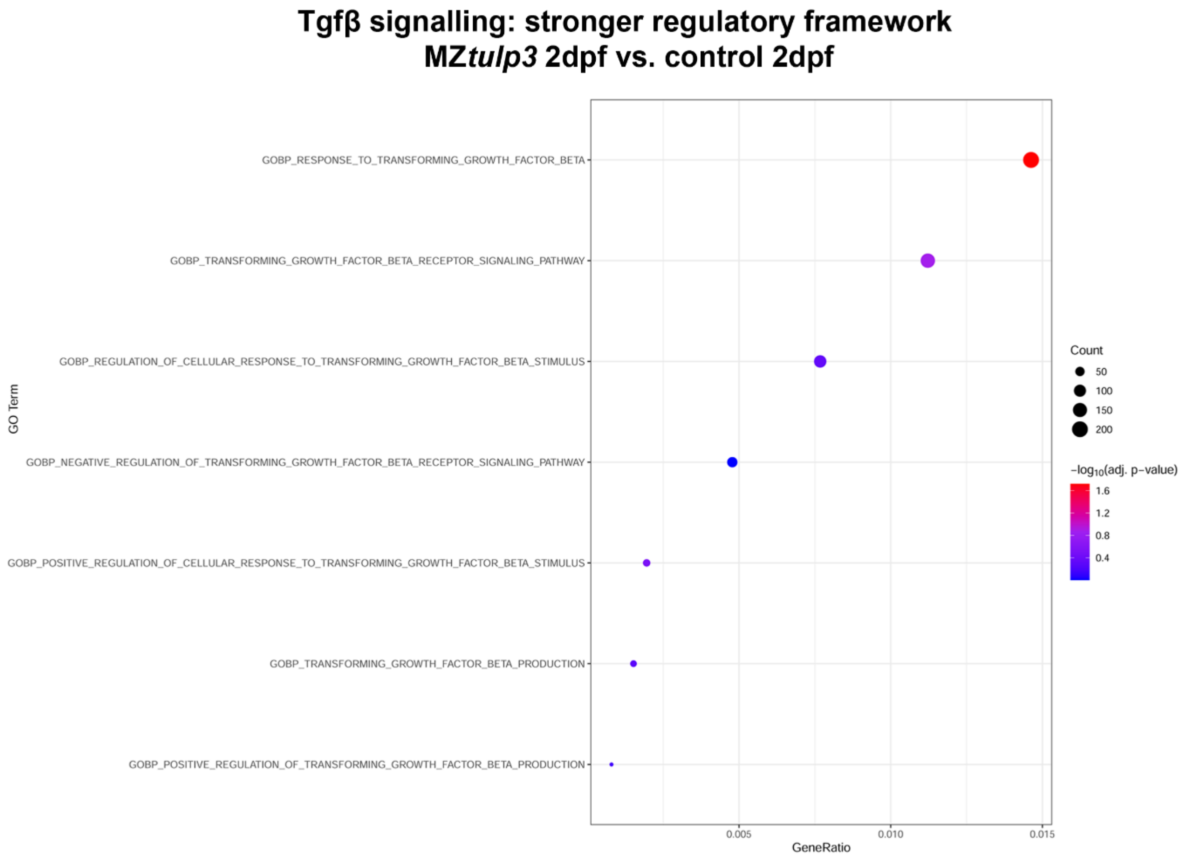

**Supplementary Figure 5**

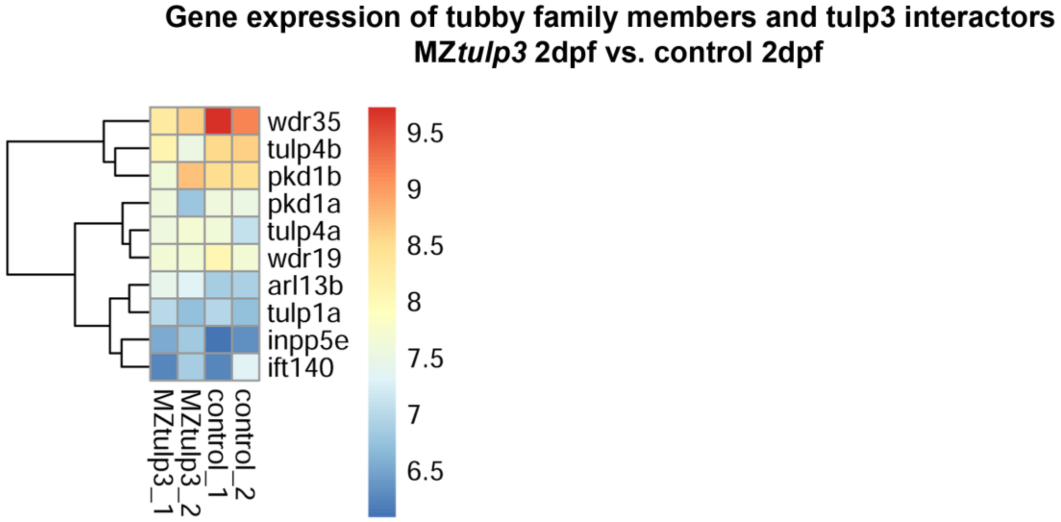

**Supplementary Figure 6**

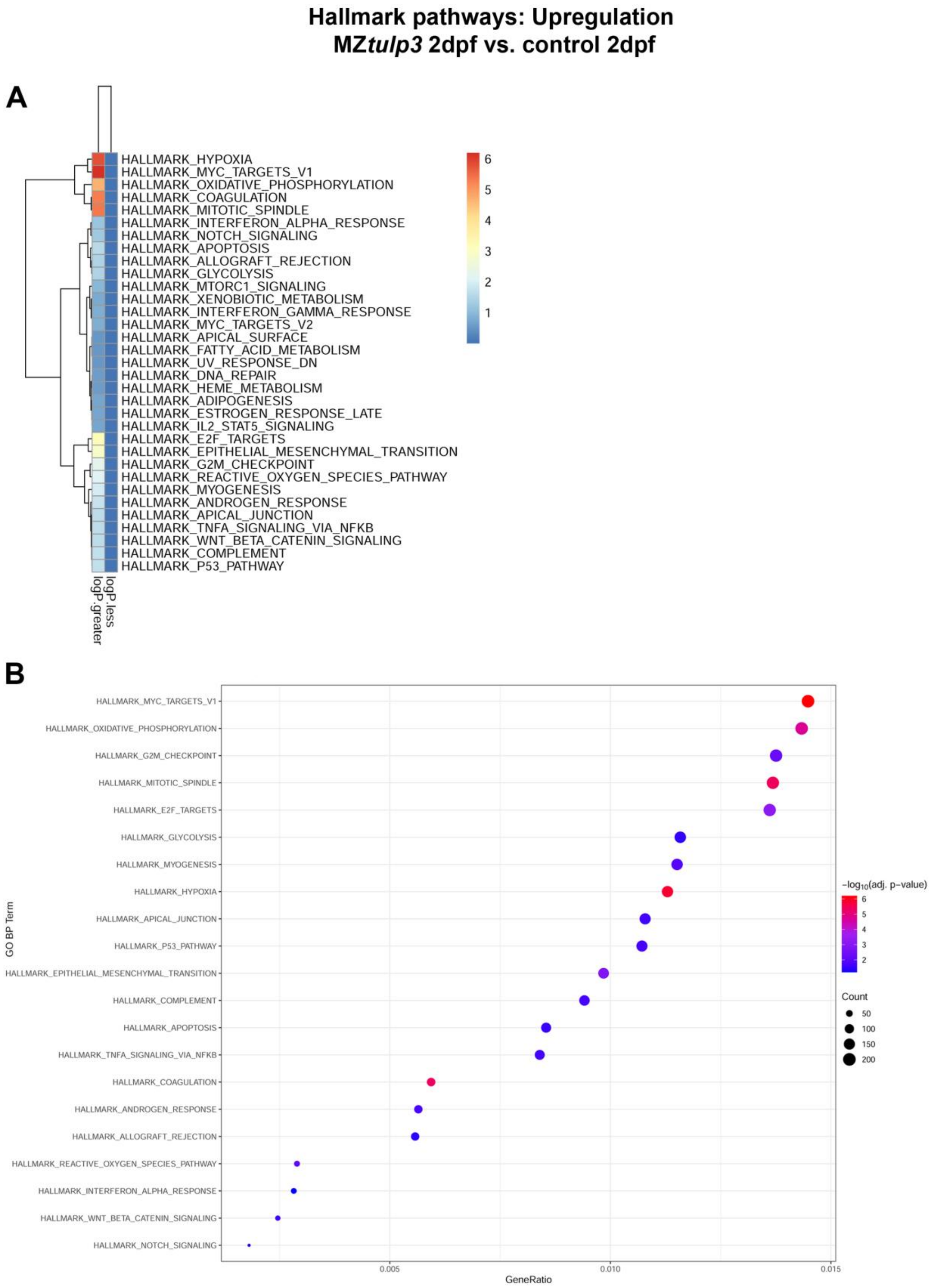

**Supplementary Figure 7**

# Fig. 1B

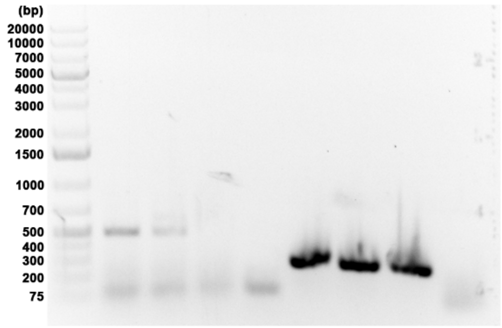


# Fig. 1C

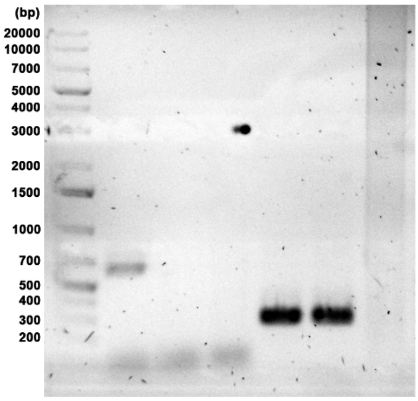

## Figure Legends

**Suppl. Fig. 1 Gene set enrichment analysis of Wnt signalling.**

Gene set enrichment analysis (GSEA) of Wnt signalling pathway associated biological processes (GOBP) in MZ*tulp3* mutant compared to control embryos at 2dpf. Pathway significance is indicated by color; pathways with Benjamini-Hochberg adjusted p-values <0.05 were considered significant.

**Suppl. Fig. 2 Differential gene expression associated with Hh signalling.**

Volcano blot of differentially expressed genes (DEGs) associated with smoothened signalling identified via GSEA. Non-significant genes are unlabeled. Significant upregulation of smoothened-related signalling genes in MZ*tulp3* was detected only in the ciliary TZ protein encoding gene *ttc23*.

**Suppl. Fig. 3 Gene set enrichment analysis of ECM-related processes.**

GSEA of ECM-related biological processes (GOBP) in MZ*tulp3* mutant compared to control embryos at 2dpf. Pathway significance is indicated by color; pathways with Benjamini-Hochberg adjusted p-values <0.05 were considered significant.

**Suppl. Fig. 4 Gene set enrichment analysis of Tgfβ signalling.**

GSEA of Tgfβ signalling associated biological processes (GOBP) in MZ*tulp3* mutant compared to control embryos at 2dpf. Pathway significance is indicated by color; pathways with Benjamini-Hochberg adjusted p-values <0.05 were considered significant.

**Suppl. Fig. 5 Gene expression heatmap of Tulp3 associated genes.**

Heatmap of tubby family members and genes encoding for known Tulp3-interacting proteins at 2dpf. Columns represent individual MZ*tulp3* and control samples.

**Suppl. Fig. 6 Analyses of oncogenic and fibrosis-related processes.**

**(A)** Heatmap of oncogenic and fibrosis-related processes in MZ*tulp3* mutant compared to control embryos at 2dpf. **(B)** GSEA of oncogenic and fibrosis-related processes in MZ*tulp3* mutant compared to control embryos at 2dpf. Pathway significance is indicated by color; pathways with Benjamini-Hochberg adjusted p-values <0.05 were considered significant.

**Suppl. Fig. 7 Respective uncropped and unprocessed gel images.**
